# Machine Learning for TCR Repertoire Epitope Annotation and Pattern Discovery

**DOI:** 10.1111/imr.70143

**Published:** 2026-07-19

**Authors:** Romi Vandoren, Vincent Van Deuren, Fabio Affaticati, Sofie Gielis, Kris Laukens, Pieter Meysman

**Affiliations:** ^1^ Adrem Data Lab, Department of Computer Science University of Antwerp Antwerp Belgium; ^2^ Antwerp Unit for Data Analysis and Computation in Immunology and Sequencing (AUDACIS) University of Antwerp Antwerp Belgium; ^3^ Antwerp Center for Translational Immunology and Virology (ACTIV), Center for Health Economics Research and Modelling Infectious Diseases (CHERMID), Vaccine and Infectious Disease Institute (VAXINFECTIO) University of Antwerp Wilrijk Belgium

## Abstract

T cells are central to adaptive immunity, recognizing antigenic peptides, called epitopes, via the T cell receptor (TCR). The immense diversity and cross‐reactivity of the TCR repertoire makes direct interpretation of antigen specificity from repertoire sequencing challenging. High‐throughput sequencing enables large‐scale profiling of TCRs but does not directly reveal their target epitopes, requiring computational approaches to bridge this gap. This review outlines two complementary strategies, bottom‐up and top‐down approaches, to annotate TCR specificity. Bottom‐up methods predict TCR‐epitope specificity from curated TCR‐epitope databases, identifying recurring patterns through distance‐based, feature‐based, or deep learning models. While effective for well‐characterized epitopes, they are limited by biased training data, absence of negative data, and weak generalization to unseen epitopes. Top‐down approaches instead infer antigen‐driven responses from repertoire‐level signals such as sequence similarity, enrichment, and TCR convergence. These methods enable discovery of disease‐ or exposure‐associated TCR signatures without prior epitope knowledge but are sensitive to technical noise and biological confounding. Both approaches are complementary as bottom‐up provides mechanistic specificity, while top‐down enables discovery in complex datasets. Their integration, alongside multimodal modeling and improved benchmarking, is key to advancing TCR‐epitope annotation and understanding adaptive immune responses.

## 1 Introduction

T cells are central to our adaptive immune system by providing protection against a wide range of external and internal threats. They do this through recognition of antigenic peptides, which initiates immune activation to eliminate infected or dangerous cells and contributes to long term immune memory. Distinct T cell subsets provide specialized roles for coordinating these immune responses, such as cytotoxic killing of target cells by CD8^+^ T cells as well as immune regulation and maintaining homeostasis by CD4^+^ T helper cells [1, 2]. These immune functions are triggered through the interaction between the T cell receptor (TCR) and antigen‐derived peptides presented by major histocompatibility complex (MHC) molecules on the surface of antigen‐presenting cells [3].

T cells recognize a variety of antigens, including peptides derived from pathogens such as viruses and bacteria, as well as overexpressed or mutated self‐antigens present in cancer or autoimmune diseases. Each T cell expresses a unique heterodimeric TCR which consists of two independent protein chains that mediate antigen recognition. The TCR interacts with antigenic‐derived peptides, also referred to as epitopes, presented by MHC class I or class II molecules [3, 4]. In humans, MHC molecules are encoded by highly polymorphic human leukocyte antigen (HLA) genes [5]. Variation in HLA alleles across individuals determines which epitopes can be presented and changes the structural context in which they are displayed. As a result, TCR recognition is inherently HLA‐restricted and the set of epitopes available for recognition differs substantially between individuals depending on their HLA genotype [6, 7, 8].

The structural mechanisms underlying TCR‐epitope binding add significant complexity to this interaction. Most TCRs consist of an independently arranged α and β chain, each containing three complementary‐determining regions (CDRs) that are involved in binding with the peptide–MHC (pMHC) complex. While the germline‐encoded CDR1 and CDR2 are mainly involved in binding the MHC molecule, the highly variable CDR3 loops of both chains interact with the antigenic peptide itself [9]. The β chain has historically been considered the most diverse chain and main determining factor for epitope specificity. However, it is now well established that both chains contribute to antigen recognition and paired αβ information often improves predicting TCR‐epitope binding probability [10, 11].

The full collection of TCRs present within an individual is called the TCR repertoire. Individual TCR sequences in the repertoire are generated during T cell development through recombination of variable (V), diversity (D) and joining (J) gene segments encoded in the genome [12]. Additional repertoire diversity arises from junctional nucleotide insertions and deletions at the V(D)J junctions and thymic selection processes that dictate the functional T cell compartment. Together, these processes generate an extremely high theoretical diversity estimated to range between 10^15^ and 10^61^ distinct TCR sequences [13]. In practice, however, the actual repertoire diversity is limited by the total number of T cells present in the body. While this is significantly lower than the theoretical diversity, it still far exceeds what can be experimentally captured from a single sample [13, 14].

In addition to its high diversity, the TCR repertoire is also characterized by redundancy in antigen recognition. A single TCR can recognize multiple distinct epitopes [15], while a given epitope can be recognized by a diverse set of TCRs [16]. This many‐to‐many relationship reflects the cross‐reactive nature of TCR‐epitope interactions and contributes to the flexibility of the adaptive immune response. At the same time, both the diversity and redundancy of the TCR repertoire significantly complicate predicting TCR‐epitope interactions from repertoire data alone [17]. To account for this complexity and be able to reliably interpret repertoire sequencing data, advanced computational approaches are required.

High‐throughput adaptive immune receptor repertoire (AIRR) sequencing allows large‐scale profiling of thousands to millions of TCR sequences. AIRR‐seq provides a snapshot of the TCR repertoire, reflecting the diversity and clonal composition within an individual at a given time point. Although this captures only a fraction of the full repertoire, the resulting TCR subsample has proven to be sufficiently representative for studying immune responses, clonal expansion and immunological history. AIRR‐seq data can reveal expanded T cell clones, that is, T cells with a unique TCR sequence, that appear in response to antigen exposure. Sometimes this includes public clonotypes that are shared between individuals exposed to the same pathogen. Such patterns in the data including clonal expansion, sequence similarity, public clonotypes and repertoire overlap have been used to identify disease‐associated TCRs, TCR cluster motifs and past exposure immune responses [18, 19, 20]. However, AIRR‐seq alone does not provide information on the epitopes recognized by these TCRs, limiting direct interpretation of antigen specificity from repertoire data.

Experimental approaches such as peptide–MHC multimers [21], activation‐based assays [22] and high‐throughput single cell technologies can provide such epitope‐specific annotations [23, 24]. However, these approaches are limited in scale and often quite expensive. Given the enormous diversity of both the TCR repertoire and epitope space, experimental mapping of all possible TCR‐epitope combinations is impossible. As a result, only a small fraction of TCR sequences have been experimentally annotated with epitope specificity and available datasets are typically biased toward well‐studied pathogens and immunodominant antigens [25].

Despite these challenges, mapping TCRs to their cognate epitopes is essential for understanding T cell immunity. It enables the identification of epitope‐specific T cell responses in infectious diseases, vaccination studies, cancer and autoimmunity. Identifying TCRs against viral, tumor, self or microbial antigens can serve as biomarkers for immune exposure and treatment response [19, 26]. By linking receptor sequences to their antigenic targets, these approaches allow functional interpretation of large‐scale repertoire data in both health and disease. However, this remains a challenging task due to the diversity of the TCR repertoire, complexity of the TCR‐pMHC interaction and limited availability of annotated TCR‐epitope pairs [25]. These factors create a major bottleneck in translating repertoire sequencing data into functional immunological insights.

**FIGURE 1:**
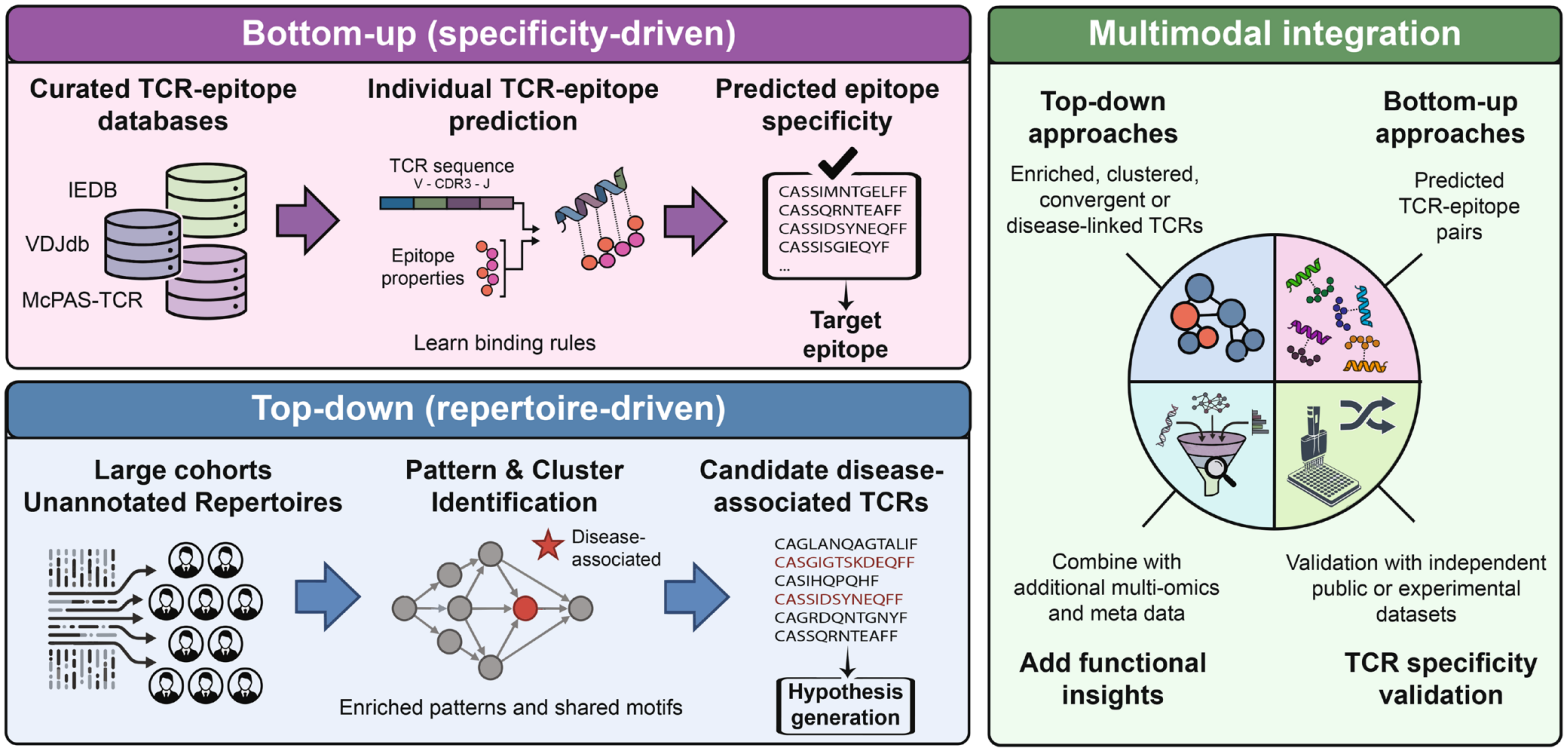
Strategic approaches for T cell receptor (TCR) specificity prediction. Bottom‐up (specificity‐driven) approaches utilize curated databases (e.g., IEDB, VDJdb, McPAS‐TCR) to learn binding rules from known TCR‐epitope pairs, enabling the prediction of TCR specificity for target epitopes. Top‐down (repertoire‐driven) approaches analyze unannotated repertoires from large cohorts to identify enriched clusters and motifs, generating hypotheses for candidate disease‐associated TCRs. Multimodal integration combines these frameworks with multi‐omics data and independent validation analyses to refine TCR specificity predictions and gain broader functional insights.

To tackle this level of complexity, computational methods have become essential to extract meaningful information from high‐dimensional repertoire data and determine TCR‐epitope specificity. Two complementary approaches have emerged (Figure 1). First, *bottom‐up* approaches start from individual TCR sequences and try to predict their epitope specificity directly. These methods learn TCR sequence features from curated databases of experimentally validated TCR‐epitope interactions and use these patterns to predict whether a given TCR recognizes an epitope of interest. They therefore work at the level of individual receptor‐epitope interactions and their associated sequence features.

In contrast, *top‐down* approaches analyze entire, unannotated repertoires to identify patterns of enriched or disease‐associated TCRs without prior knowledge of their cognate epitopes. Rather than predicting the specificity of individual TCRs for epitopes directly, top‐down methods look for statistical signals such as expansion, convergence or enrichment within specific cohorts. These signals can indicate T cell activation and reveal shared immune responses across individuals, even when the underlying epitopes driving these patterns are unknown.

Thus, bottom‐up approaches are database‐driven and specificity‐oriented, trying to assign epitope specificity to individual TCRs, while top‐down approaches are repertoire‐driven and discovery‐oriented, seeking to identify antigen‐driven patterns within TCR repertoires at the population or cohort level. Although they are conceptually different, these strategies are clearly complementary to each other since bottom‐up provides mechanistic insights into individual TCR specificity while top‐down identify candidate disease‐associated TCRs within large and heterogenous repertoires.

Despite significant progress over the last years, fundamental challenges remain that limit the interpretation and reliability of TCR‐epitope prediction models. This includes, but is not limited to, TCR cross‐reactivity [15], the size of current available training data and limited availability of paired αβ sequencing data [27] as well as the difficulty of defining or creating reliable negative training data [28], all of which can influence model performance. In addition, the limited number of validated TCR‐epitope interactions is inherently biased toward well‐known pathogens and β‐chain TCRs. Therefore, models trained on this data often struggle to generalize their predictions to other epitopes [29, 30]. Overcoming these obstacles and finding more generalizable representations of TCR‐epitope interactions will require integrated strategies that combine bottom‐up and top‐down approaches, along with standardized datasets and robust evaluation frameworks.

This review synthesizes the rapidly evolving landscape of computational approaches for TCR‐epitope annotation. We first discuss bottom‐up prediction strategies that infer antigen specificity from curated TCR‐epitope databases, followed by top‐down approaches that identify antigen‐driven TCR patterns and motifs within repertoires from large cohorts. We highlight recent methodological advances, discuss key limitations and confounding factors, and illustrate how these approaches can be integrated to improve our understanding of antigen‐specific T cell responses. Throughout, we reflect on recent contributions from our group and others in the field, focusing on approaches that bridge bottom‐up and top‐down methodologies and provide new insights into current challenges and opportunities for improvement.

## 2 Bottom‐Up Approaches

Bottom‐up approaches try to identify antigen specificity of individual TCRs by learning patterns from experimentally validated TCR‐epitope interactions. These methods focus on the level of individual receptor‐epitope pairs and attempt to map TCR sequence features directly to epitope recognition. However, this prediction task is complicated by limitations in data availability, data quality, and the complexity of the TCR‐pMHC interactions.

### 2.1 Training Datasets and Data Biases

#### 2.1.1 Positive Training Data

Most bottom‐up models are trained on curated databases such as VDJdb [31], McPAS‐TCR [32] and IEDB [33], which contain experimentally validated TCR‐epitope interactions. These are typically derived from peptide–MHC multimer assays, activation‐based assays and high‐throughput single cell techniques. The validated pairs provide positive training data needed for supervised learning to extract binding‐specific patterns within the TCR and epitope sequences. One important limitation is that the available data only represents a small and highly biased subset of the actual full TCR‐epitope landscape. Additionally, the majority of known TCR‐epitope pairs correspond to well‐characterized, immunodominant viral epitopes, while other much rarer epitopes such as tumor neoepitopes and microbiome‐derived epitopes remain underrepresented [25, 27]. Most databases are dominated by β chain sequences, as historically it has been the dominant focus of TCR research. While paired αβ data does exist, it remains relatively scarce. This limits the ability of models to capture the full determinants of antigen recognition and contributes to the incomplete representation of TCR specificity characteristics [11]. As a result, bottom‐up models are typically trained on uneven datasets with large differences in epitope and TCR chain coverage which can lead to biased predictions and reduced generalizability.

The annotation quality and reliability of the TCR‐epitope interactions are often variable. Factors like experimental noise, assay‐specific biases, and inconsistent standards can introduce false positives or ambiguous TCR‐epitope pairs. Due to the intrinsic nature of these biases, they are undeniably tied to any training data set and therefore directly influence model accuracy and performance. Taken together, the database size as well as data distribution and reliability are limiting factors for the development of robust and accurate TCR‐epitope prediction models.

Current public and private efforts aim to better curate databases by filtering low‐confidence entries, standardizing epitope annotations, removing duplicates and non‐standard sequences, and prioritizing experimentally validated interactions, thereby reducing noise and improving reliability.

#### 2.1.2 Negative Training Data

Another factor complicating bottom‐up modeling is the lack of true negative data. While positive data can be extracted from curated databases of experimentally validated TCR‐epitope interactions, negative data is much harder to find. It remains largely unknown which TCRs do not bind a given epitope. As a result, there is no comprehensive ground truth negative dataset, meaning negative training data must be artificially generated.

In general, two main strategies were developed to approximate negative data. The first approach relies on using a reference dataset, where “random negatives” are created using TCRs sampled from unrelated repertoires [28]. These new TCRs are paired with the epitopes of interest under the assumption that there is a very low probability that a random TCR would recognize the target epitope. However, especially for highly prevalent and immunodominant epitopes, this can introduce false negative results. This strategy also risks introducing dataset‐specific biases, allowing models to distinguish between positive and negative interactions based on underlying distributional differences rather than true binding determinants [28]. As a consequence, models may achieve high performance while failing to capture the biological basis of TCR‐epitope recognition.

The second strategy uses “shuffled negatives,” in which TCRs and epitopes from the positive training data are randomly reassigned to each other [28]. Compared with random sampling from background repertoires, the positive and negative samples are drawn from the same space, preventing the model from shortcut learning on distribution differences to distinguish binding from non‐binding epitopes. However, shuffled negatives can also not be considered true negatives. Due to the cross‐reactive nature of TCR recognition, a receptor known to bind one epitope may still recognize another epitope present in the dataset, particularly when epitopes share sequence, structural, or HLA‐related characteristics. As a result, both random and shuffled negative generation strategies can introduce false negatives and only imperfectly approximate non‐binding TCR‐epitope interactions in vivo.

The absence of reliable negative training data thus complicates the training and interpretation of bottom‐up prediction models and contributes to variability across studies. New methods need to be developed for the creation of biologically meaningful negative data to improve the development of robust and generalizable TCR‐epitope prediction models.

### 2.2 Encodings for TCR and Epitope Sequences

**FIGURE 2:**
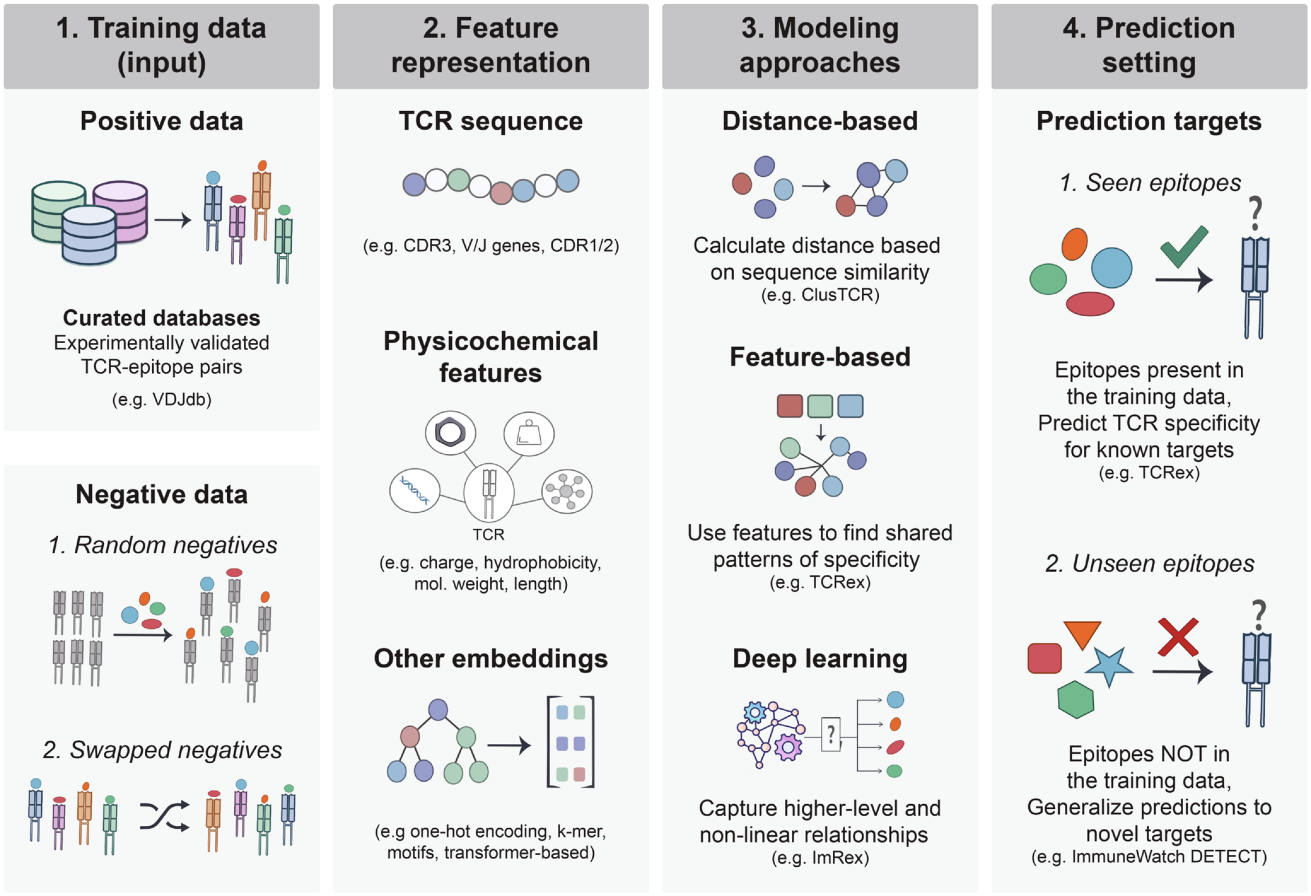
Computational workflow for bottom‐up TCR specificity prediction. (1) Training data: Models utilize positive data from curated databases and generate negative data via random sampling or swapping. (2) Feature representation: TCRs are encoded using sequence‐based information (CDR loops, V/J genes), physicochemical properties (charge, hydrophobicity), or numerical embeddings (k‐mers, transformers). (3) Modeling approaches: Strategies include distance‐based clustering, feature‐based pattern recognition, and deep learning to capture nonlinear relationships. (4) Prediction setting: Models are applied to predict specificity for seen epitopes (present in training) or generalize to unseen epitopes (novel targets).

The representation of TCR and epitope sequences plays an important role in model architecture and performance, as it defines how biological information is translated into a computationally usable feature (Figure 2). Most approaches focus on the CDR3 sequence of the β chain, as this region is responsible for the primary contact with the epitope and shows the highest sequence diversity [11]. However, restricting the representation to this region alone neglects additional structural and germline‐encoded information present in the rest of the receptor. To address this, many models include V‐ and J‐gene usage, which provides indirect structural context, or they extend the representation to include CDR1 and CDR2 regions to represent the interaction with the MHC molecule. When available, paired αβ chain information further improves predictive performance by capturing the combined contribution of both chains to epitope binding and recognition [30].

Different encoding strategies have been developed to represent these sequences. Simple one‐hot encodings treat amino acids or genes as independent categorical variables with minimal complexity but lack the ability to capture relationships between residues. Physicochemical encodings include properties such as hydrophobicity, charge, and molecular weight to embed biologically meaningful features into the model [34, 35]. K‐mer based approaches capture local sequence motifs [36], while more recent methods can also rely on protein language models and transformer‐based embeddings that learn contextual representations from large scale sequence data [37]. These embeddings can better capture long range dependencies and hidden structural features, providing a richer but more complex representation of the full TCR sequence interactions.

An important distinction between the bottom‐up models can be made based on whether the epitope sequence itself is included in the representation or not. Some approaches treat predicting TCR specificity as a simple classification problem based solely on the TCR features, assuming that epitope‐specific patterns can be learned independently of epitope features [34]. In contrast, interaction‐based models include both the TCR and epitope sequence enabling the model to learn features of their joint interaction [27]. This distinction has important implications for generalizability as models that incorporate epitope information are better positioned to capture transferable interaction patterns. Therefore, they perform more robustly when predicting specificity for epitopes not seen in the training data before (i.e., unseen epitopes). More broadly, the choice of representation format reflects a trade‐off between interpretability, bias and the ability to capture complex higher order biological relationships.

### 2.3 Modeling Approaches for Bottom‐Up Prediction

A range of modeling strategies has been developed that differ in their underlying machine learning method, how they represent the sequences, extract features, and how they handle the data limitations (Figure 2).

#### 2.3.1 Distance‐Based and Clustering Approaches

The simplest approaches are distance‐based methods, which rely on the assumption that TCRs with similar sequences are likely to recognize the same epitope [17]. These models often focus on the CDR3 loop of the α and/or β chain and quantify similarity using sequence‐based metrics, such as edit distance, which represents the number of residues that differ between sequences or more specialized measures. Clustering algorithms can then be applied to group similar TCRs together based on pairwise similarity and enrichment of shared motifs. However, these approaches do not include epitope sequence information in the predictions. Instead, epitope specificity is assigned based on epitope annotations of similar sequences. Current methods like ClusTCR [38] and TCRdist [20] calculate sequence distance and cluster TCRs based on their sequence similarity and motifs to identify antigen‐driven patterns.

Such distance‐based or clustering approaches perform well when TCRs recognizing the same epitope cluster closely together in the sequence space. They require minimal to no training data, are robust to small dataset sizes, and provide easily interpretable results. However, using simple distance metrics to decide epitope specificity is limited in its ability to capture more complex or non‐linear relationships and can fail when epitope‐specific TCRs are highly diverse in sequence.

#### 2.3.2 Feature‐Based Machine Learning Models

To capture more complex patterns beyond simple sequence similarity, feature‐based machine learning models are used to identify shared patterns that underlie TCR‐epitope specificity. These models learn associations between TCR sequence features and epitope specificity using labeled positive and negative datasets. They can use a variety of feature representations, including CDR3 sequence motifs [35, 36], V‐ and J‐gene usage, and physicochemical properties [34, 35], represented using various encoding strategies. Feature‐based approaches can be used in an epitope‐specific setting, where separate models are trained for individual epitopes [34]. In contrast, in a pan‐specific setting, a single model will predict specificity across multiple epitopes simultaneously [39]. While epitope‐specific models often achieve high performance for individual well‐characterized epitopes, pan‐specific models aim to capture more generalizable patterns across the broader antigen spectrum.

#### 2.3.3 TCRex as a Feature‐Based Prediction Model

Classical machine learning algorithms such as random forests are particularly suited for predicting epitope specificity. They can model non‐linear relationships between the TCR and epitope, can handle heterogenous feature types, and are relatively robust to noise in the data. An example of this is TCRex [34], a random forest model developed in our lab, used for predicting TCR specificity against known epitopes. TCRex uses physicochemical representations of the CDR3 region combined with one‐hot encoding of the V‐ and J‐gene usage to train epitope‐specific classifiers. TCRex was trained on positive training data derived from manually curated pathology‐associated T cell receptor sequences (McPAS‐TCR [32]), the VDJ database [31], the ImmuneCODE database [40] and TCR‐epitope interactions found in the literature. Negative data was created by randomly sampling from a large number of unrelated TCR repertoires derived from healthy individuals [41].

For each of the currently 100 different epitopes (93 viral and 7 cancer) within the TCRex training data, a separate random forest model is trained. For every TCR in the repertoire, the model returns the binding prediction score between the TCR and epitope and a baseline prediction rate value. This value reflects how many TCRs in a background/naive repertoire are expected to have a score equal to or higher than the TCR of interest. Thus, TCRex performs enrichment analysis to determine whether TCRs against specific epitopes are overrepresented within a repertoire, as compared to a background distribution. A user‐defined threshold allows limiting the number of false positive hits.

One important characteristic of TCR‐epitope interactions is that a single epitope can be recognized by multiple different TCR motifs. The architecture of random forest models is particularly suited to capture this heterogeneity. These models can learn multiple independent decision boundaries rather than being forced to converge on a single dominant TCR pattern. In addition, their robustness to noise makes them well suited for biological datasets that are known to contain mislabeled data and experimental errors. This explains why TCRex has shown a strong performance compared to other more complex modeling techniques [10].

The functional value of the TCRex models was shown in both cancer and viral disease studies. In acute myeloid leukemia (AML), the combination of clustering analysis with ClusTCR and specificity prediction through TCRex revealed a potential link between the treatment response in patients and the diversity of the epitope‐specific TCR repertoire [42]. Here, TCRex models allowed the identification of TCRs specific for two Wilms' tumor protein 1 (WT1) epitopes, WT1‐37 and WT1‐126, in the repertoires of AML patients treated with hematopoietic stem cell transplantation. By integrating sequence similarity‐based clustering with feature‐based specificity predictions, this approach revealed that the repertoires of patients showing complete response demonstrated more diverse TCR patterns within the WT1‐specific clusters compared to relapsing patients. This suggests that the diversity of antigen‐specific TCR responses may contribute to protection against relapse. The study also demonstrates that epitope‐specific TCR repertoires can be tracked directly from bulk repertoire sequencing data, providing a non‐invasive biomarker for monitoring immune responses and therapy outcomes. Together, these findings illustrate how bottom‐up approaches can uncover clinically relevant links between the T cell repertoire and treatment response.

#### 2.3.4 Deep Learning Models

More recently, deep learning approaches have been introduced to capture higher level interactions within the TCR‐pMHC complex by simultaneously modeling structural, biochemical, and nonlinear relationships. Model architectures such as convolutional neural networks and transformer‐based models can integrate multiple input modalities including paired αβ chains, extended TCR sequence context, and in some cases also epitope information [27, 43]. For example, protein language models treat TCR sequences as a form of biological language enabling the extraction of contextual embeddings that capture residue dependencies and structural features [44].

Despite these advantages, the performance of deep learning models in TCR‐epitope prediction is often affected by the limited size and quality of available training data. These models are inherently data‐hungry and may struggle to generalize in settings where labeled examples are scarce or biased. In practice, simpler models such as distance‐based approaches or random forests can achieve comparable performance to deep learning, particularly for well‐characterized epitopes, highlighting that increased model complexity does not necessarily translate into improved predictive accuracy.

Furthermore, deep learning models often operate as “black boxes”, limiting interpretability and making it difficult to extract biologically meaningful insights from their predictions. While techniques such as attention mechanisms and feature attribution methods can provide some interpretability [43], these remain less intuitive compared to motif‐based or feature‐based approaches. Current efforts therefore increasingly focus on combining deep learning with pretraining strategies and biologically informed representations to improve generalization while maintaining interpretability.

### 2.4 Seen Versus Unseen Epitopes

Bottom‐up approaches can be applied in either a “seen” or “unseen” epitope setting [30, 45]. In the case of seen epitopes, the target epitope is already present in the training data, allowing models to learn epitope‐specific patterns directly from labeled TCR‐epitope pairs. The prediction task then consists of determining whether a given TCR sequence is likely to recognize a known epitope based on its learned patterns. The AML study [42] described above is an example of this seen epitope setting as predictions were performed for epitopes included in the TCRex training dataset. When sufficient and high‐quality training data are available, models can achieve strong predictive performance for seen epitopes. However, this setting is limited to only epitopes for which experimental data exists.

In contrast, predicting specificity for unseen epitopes represents a substantially greater challenge. In this setting, no labeled TCR‐epitope pairs exist for the target epitope, thus the model must rely on generalizable features learned from other interactions. This shifts the task from memorizing epitope‐specific sequence patterns to capturing underlying principles of TCR‐pMHC recognition. From a machine learning perspective, several factors contribute to the difficulty of this task. First, sequence similarity between epitopes does not necessarily translate into similarity in TCR recognition, as structurally distinct peptides can adopt similar conformations within the MHC binding groove, and small sequence changes can have disproportionate effects on TCR binding [46]. Second, TCR recognition is highly dependent on the structural context provided by the MHC molecule, introducing an additional layer of variability that is often not explicitly modeled [6]. Third, the inherent cross‐reactivity of TCRs means that a single receptor can recognize multiple unrelated epitopes [15]. These factors complicate the identification of transferable binding rules that can be generalized from training data to previously unseen epitopes.

Importantly, the distinction between seen and unseen epitopes is not strictly binary. A range exists from TCRs with similar sequence or structural features to entirely novel TCR‐pMHC combinations [46]. Model performance typically degrades gradually as TCRs become less similar, reflecting the extent to which learned features can be transferred. Current benchmarking efforts consistently demonstrate a drop in performance when moving from seen to unseen epitopes, highlighting the limitations of existing models [10, 30, 45]. Addressing this challenge will require approaches that incorporate interaction‐level representations and model the underlying biological principles of TCR‐epitope recognition.

### 2.5 Models That Can Generalize Across Different Epitopes

To address the current limitations of epitope‐specific models and the lack of transferable patterns, new efforts have been focused on the development of approaches that are able to generalize across epitopes. Such models could help improve transferability of predictions as they learn shared patterns from diverse TCR‐epitope interactions to be applied on potential target epitopes, potentially including unseen epitopes.

#### 2.5.1 ImmuneWatch DETECT as Pan‐Specific Model

One such approach is the ImmuneWatch DETECT framework [39], which combines probabilistic modeling with motif‐based clustering to assign epitope specificity. DETECT uses a pan‐specific epitope prediction model and builds on many of the aspects that made TCRex successful. The core annotation principle is probabilistic, where it will evaluate for any TCR whether sufficient information is present to assign an epitope label. It also uses common clustering principles to define different motifs for each epitope. In this manner, every input TCR is not evaluated for a single epitope, but for all epitopes that might have a motif match. A local feature‐based prediction model is then used to score the most probable binder epitope based on the strength of the motif signal compared to all other candidate epitopes. This uses the information collected about other epitopes to strengthen its signal, so that the final score enumerates the specificity of the input TCR with the provided epitope, resulting in a true pan‐specific model. Finally, a major advantage is that DETECT is built on the proprietary ImmuneWatch database, which is larger than public collections, improving the prediction results. By leveraging this large dataset of annotated interactions, DETECT can identify epitope‐specific motifs while maintaining flexibility across different epitope contexts. Results from the 2023 IMMREP benchmarking effort [30] showed that ImmuneWatch DETECT had the best performance among the models, both for seen and unseen epitopes followed by models from the Qimmuno lab, MixTCRpred [47] and NetTCR 2.2 [29].

An important application of pan‐specific annotation models was recently demonstrated in an analysis of TCR repertoires from multiple sclerosis (MS) patients and controls [48]. By applying ImmuneWatch DETECT to integrated public TCR datasets, we were able to annotate epitope specificities across entire repertoires and systematically test for viral enrichment patterns. Our study revealed a significantly increased frequency of Epstein–Barr virus (EBV)‐specific TCRs in MS patients, particularly within the CD8^+^ T cell compartment, with no such enrichment observed for cytomegalovirus (CMV) or other viral responses. Interestingly, the study also showed a decreased frequency of myelin‐reactive TCRs in the MS repertoires compared to controls when looking at sequence similarity‐based matching, alongside the depletion of several public TCR clusters, several of which were associated with CMV exposure. These findings highlight that EBV‐specific immune responses might cause complex dysregulation of the immune system in MS. Such insights would be difficult to obtain using the more basic epitope‐specificity models as they require prior knowledge of target epitopes and do not scale well to full repertoire analysis. In contrast, pan‐specific frameworks like DETECT enable hypothesis‐free annotations across diverse epitopes making it possible to uncover broad and unexpected immunological patterns such as virus‐autoimmunity links and molecular mimicry mechanisms. This highlights the biological relevance of such models in translating TCR repertoire data into disease‐associated immune signatures.

#### 2.5.2 ImRex to Include Both Interaction Partners

Another way to complement current approaches and move beyond single sequence TCR predictions is to model both the TCR and epitope sequence and their higher‐level interactions. Therefore, our lab developed ImRex (interaction map recognition), designed to explicitly model TCR‐epitope interactions for seen and unseen epitope specificity. ImRex is an interaction‐focused convolutional neural network (CNN) inspired by image‐processing architectures [27, 43]. It converts TCR CDR3 and epitope sequences into interaction maps by computing pairwise differences between selected physicochemical properties of their amino acids, including hydrophobicity, hydrophilicity, mass, and isoelectric point. Each physicochemical property corresponds to a separate channel, resulting in a multi‐channel representation analogous to an image.

These interaction maps are then processed by a multi‐layer CNN to predict TCR‐epitope binding. By explicitly combining information from the physicochemical features of both the TCR and the epitope sequence, ImRex learns higher‐level representations of their interaction context rather than treating TCRs and epitopes independently. Because it is trained on diverse publicly available TCR‐epitope pairs, the model is forced to identify transferable physicochemical interaction patterns that generalize across different peptide contexts. This design reduces reliance on single epitope‐specific patterns and helps prevent overfitting, making ImRex particularly suited for the prediction of unseen epitopes.

#### 2.5.3 The Next Step in Bottom‐Up TCR‐Epitope Predictions

Together, these bottom‐up approaches illustrate complementary strategies for solving the TCR‐epitope prediction problem. TCRex represents a class of epitope‐specific models that perform well in the seen epitope setting and are well‐suited for rapid, targeted screening of known antigens. In contrast, pan‐specific frameworks like DETECT address both the seen and unseen epitope setting by enabling repertoire‐wise and hypothesis‐free annotation across a broad range of epitopes. It is further supported by its large annotated database and the use of probabilistic modeling. Complementary to these, the interaction‐based modeling from ImRex encodes physicochemical relationships between the TCR and epitope sequences, providing a mechanistic higher‐level approach to capturing the determinants of binding. Therefore, it is better suited to generalize beyond individual epitopes by learning the underlying biological mechanisms facilitating TCR‐epitope recognition. Both DETECT and ImRex allow expanding predictions to poorly characterized or novel epitopes.

Rather than competing alternatives, these model classes address different aspects of the same problem space and form a continuum from epitope‐specific predictions to broad, discovery‐oriented analyses. By combining insights from each section and as datasets continue to grow, current approaches enable the exploration of full TCR repertoires in terms of antigen specificity, which will become integral to both immunological diagnostic research and clinical immune monitoring.

### 2.6 Benchmarking and Improving Future Models

As the number of machine learning models for TCR‐epitope prediction has increased rapidly, standardized benchmarking efforts have become essential for comparing performance. The IMMREP challenges (2022, 2023, and 2025) provided a structured framework for evaluating TCR specificity prediction across model architectures and prediction settings, including both seen and unseen epitopes.

These benchmarks revealed several patterns in model performance. Models incorporating paired αβ chain information generally outperformed the single‐chain approaches, indicating that both chains provide complementary information for predicting TCR‐epitope interactions. Similarly, including additional sequence context such as V‐ or J‐gene usage and CDR1/2 regions alongside the CDR3 region further improved predictive performance [10, 30]. Overall, these results suggest that feature‐based, epitope‐specific models integrating multiple sources of sequence information can provide a strong balance between predictive performance, robustness, and interpretability in the seen epitope setting.

Importantly, benchmarking also highlighted the substantial performance gap between seen and unseen epitope prediction, as well as the strong influence of dataset composition and evaluation strategy on reported results [30, 45]. These findings emphasize that model performance must be interpreted in the context of the underlying data and task definition. Currently, there is a strong need for more standardized, carefully curated datasets, consistent evaluation protocols, and continued community efforts to improve comparability and reproducibility across studies.

Overall, while bottom‐up TCR prediction models, including distance‐based, feature‐based and neural network approaches have demonstrated promise, their accuracy and generalizability are constrained by their reliance on experimentally annotated TCR‐epitope interactions. As a result, their applicability is limited in settings where little or no prior knowledge of antigen specificity is available. This is particularly relevant for complex diseases, heterogeneous immune responses and novel antigens, where the epitope landscape is incompletely characterized. These limitations motivate the development of complementary strategies that do not depend on predefined specificity labels but instead infer antigen‐driven signals directly from repertoire‐level patterns. This forms the basis of top‐down approaches, which shift the focus from individual receptor‐epitope pairs to the collective structure and composition of TCR repertoires to identify population‐level signatures associated with antigen exposure, disease state, or other phenotypic traits.

## 3 Top‐Down Approach

**FIGURE 3:**
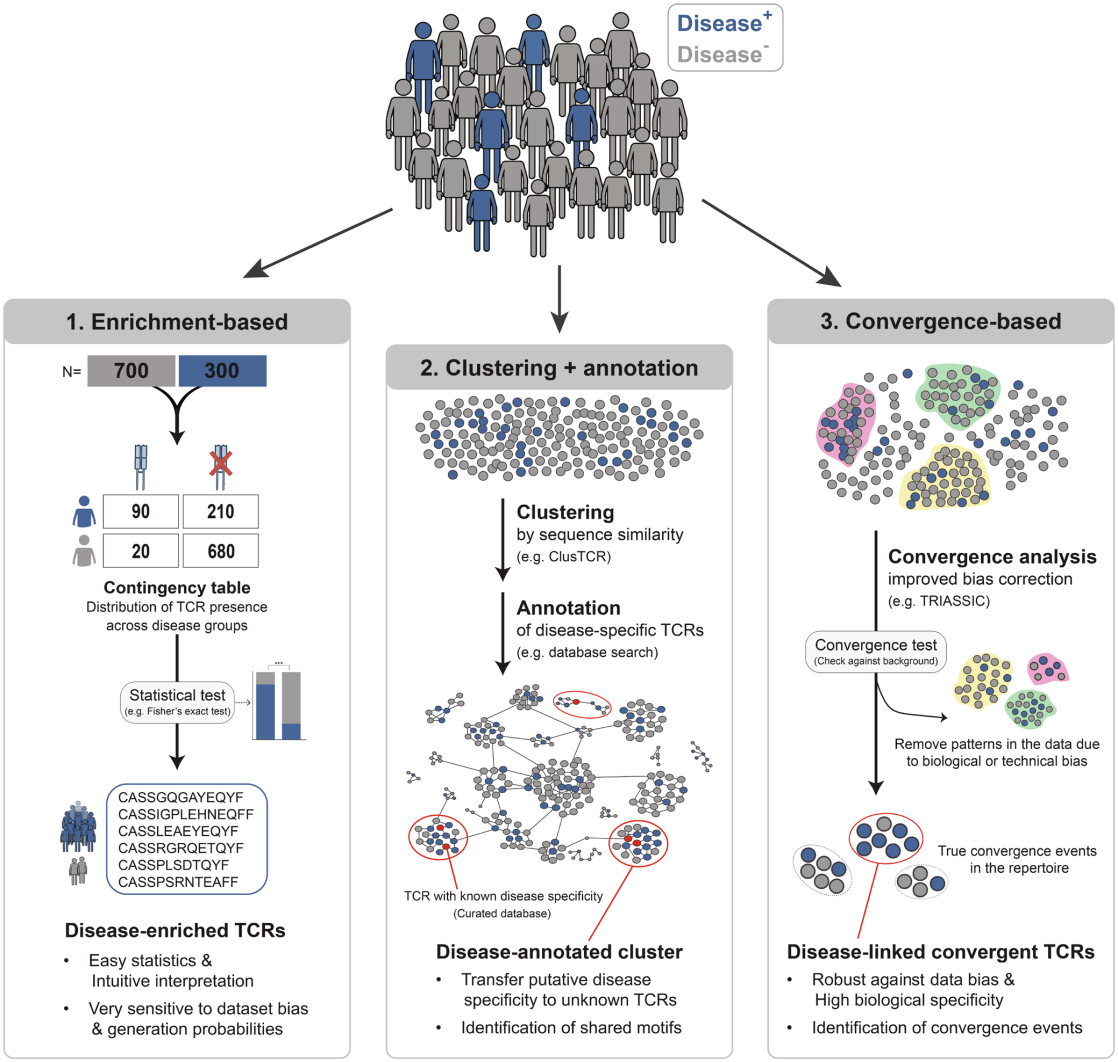
Top‐down strategies for identification of disease‐associated TCR patterns. This repertoire‐level approach leverages TCR repertoire data from large cohorts to discover disease‐relevant signals without requiring pre‐existing epitope annotations. (1) Enrichment‐based methods identify individual TCRs statistically overrepresented in a target condition using statistical analyses. (2) Clustering followed by annotation identifies sequence‐similar TCRs, enabling known annotations to be transferred to putatively epitope‐ or disease‐specific clusters. (3) Convergence‐based methods utilize bias correction and statistical tests against background models to distinguish biologically relevant expanded, disease‐convergent TCRs from data noise.

Top‐down approaches build on the idea that antigen‐driven T cell responses leave detectable imprints at the level of the TCR repertoire. Rather than relying on experimentally validated TCR‐epitope pairs, these methods analyze large‐scale repertoire data to detect patterns that are indicative of shared immune responses across individuals or conditions. The underlying principle is that recognition of their cognate antigen leads to expansion of specific T cell clones, resulting in detectable changes in clonal frequency, sequence similarity patterns and repertoire composition. By identifying such deviations from baseline repertoire structure, top‐down approaches aim to detect TCRs and clusters that are likely involved in shared immune responses, even without explicit epitope annotations (Figure 3, Table 1).

**TABLE 1:** Overview of computational methods for TCR specificity and repertoire analysis.

Category	Method	Key principle	Strengths	Limitations	Examples
Bottom‐up	Distance‐based/clustering	Sequence similarity	Interpretable; no/low training; robust to small datasets	Misses non‐linear patterns; poor for diverse repertoires; no epitope modeling	ClusTCR [38]
	Feature‐based	Learns sequence features predictive of epitope specificity	Handles heterogeneity; robust; interpretable; strong for seen epitopes	Needs curated positives/negatives; limited transferability	TCRex [34]
	Deep learning	Learns non‐linear sequence features; Complex modeling of TCR‐epitope interaction features	Captures complex interactions; flexible input integration; better generalization to unseen epitopes	Data‐hungry; overfitting risk; limited interpretability	ImRex [27]
	Pan‐specific models	Learns shared motifs across epitopes; probabilistic annotation	Scales to full repertoires; works for seen/unseen epitopes	Dependent on database size/quality; less mechanistic	ImmuneWatch DETECT [39]
Top‐down	Enrichment‐based	Identify TCRs enriched in condition (e.g., disease, infection)	No prior annotation needed; strong biomarker potential	Sensitive to confounding; requires large cohorts	Statistical testing [49]
	Clustering‐based	Group similar TCRs to detect expanded antigen‐driven clusters	Increases statistical power; captures motif‐level signals	No direct specificity; requires downstream annotation	ClusTCR
	Convergence‐based	Detect convergent TCRs due to antigen‐driven selection	Combines enrichment with strengths from clustering; robust to technical and biological bias	Computationally complex; indirect specificity	TRIASSIC [50]
Hybrid/integrative	Multimodal integration	Integrates repertoire with functional/contextual data	Links specificity to phenotype and environment; higher level interpretation	Requires multiple data modalities; complex data integration	STEGO [51], AIRRWAS [52]

*Note:* Summary of methodological categories, highlighting their underlying principles, relative technical advantages, inherent limitations and representative software examples.

These methods therefore leverage population‐scale data to discover antigen‐associated signals that might not be identifiable through direct TCR‐epitope predictions if the relevant antigens are unknown or not experimentally characterized. This makes them particularly well suited for studying complex diseases and heterogenous immune responses.

### 3.1 Enrichment‐Based Approaches

A first class of top‐down approaches focuses on identifying individual TCR sequences that are shared between individuals (public clonotypes) or significantly enriched in specific cohorts. These methods typically rely on statistical comparisons between groups (e.g., diseased vs. healthy individuals) to detect TCRs that occur more frequently than expected by chance. Enrichment‐based strategies have been successfully applied in large‐scale cohort studies (repertoires from 600 to 3000 individuals), where epitope‐specific TCRs can be extracted directly from whole repertoires, for example, in the context of cytomegalovirus (CMV) infection.

An important example is the Emerson CMV study [19], in which peripheral blood TCRβ repertoires from hundreds of individuals with known CMV serostatus were analyzed to identify CMV‐associated TCR sequences. In this study, the authors applied a statistical enrichment framework to compare the frequency of individual TCR sequences between CMV‐positive and CMV‐negative individuals. By performing enrichment testing across the cohort, they identified a set of public TCRβ sequences that were significantly overrepresented in CMV‐positive individuals. These sequences were interpreted as signatures of CMV‐specific immune responses, despite the fact that their cognate epitopes were not directly annotated. Importantly, the identified TCRs were then used to train a classifier that could predict CMV serostatus in independent cohorts with high accuracy, demonstrating that repertoire‐level signals can be sufficiently robust to capture antigen exposure history.

These findings are supported by work from our group, in which the transferability and robustness of the CMV‐associated TCR signatures were evaluated in an independent cohort [49]. In this study, TCRβ repertoires from a Belgian population were analyzed with a focus on the CD4^+^ memory compartment. Previously identified CMV‐associated TCR sequences were used to assess whether enrichment‐based signals could be reproduced across populations and experimental conditions. A substantial fraction of these sequences could be detected in the independent dataset, indicating that they represent shared immune responses rather than cohort‐specific artifacts. Moreover, these CMV‐associated TCRs were present within the CD4^+^ memory repertoire, supporting their persistence as part of long‐term immunological memory. Applying the same statistical framework enabled accurate prediction of CMV serostatus, with performance comparable to that observed in the original study. Together, these results demonstrate that enrichment‐based repertoire signals can be robust across populations, sequencing strategies and T cell subsets.

These studies illustrate several key principles underlying enrichment‐based top‐down approaches. First, antigen exposure can leave a detectable imprint on the global TCR repertoire through clonal expansion and increased sharing of antigen‐specific receptors across individuals. Second, statistical association at the population level can be used to infer antigen specificity indirectly, even in the absence of explicit TCR‐epitope annotations. Finally, the reproducibility of these signals across independent cohorts highlights their potential for biomarker discovery and immune monitoring. This shows that top‐down methods are powerful because they require no prior epitope annotation, yet they are also highly dependent on sequencing protocol, sequencing depth, repertoire size and batch effects, and thus require large and well‐controlled cohorts.

### 3.2 Sequence Similarity and Clustering

Clustering‐based approaches represent another key strategy within top‐down repertoire analysis. While clustering can be used in combination with bottom‐up methods (as illustrated in the AML example), it can independently serve as a top‐down method by identifying groups of similar TCRs directly from the full repertoire. To overcome the sparsity of exact sequence matches, these methods group TCRs based on sequence similarity and shared motifs (e.g., using tools like ClusTCR [38]). TCR clusters are created since TCRs recognizing the same epitope often share conserved patterns or structural characteristics even if their sequences are not identical. By aggregating similar TCRs, clustering‐based methods increase statistical power and enable the detection of shared immune responses across individuals.

In the top‐down setting, clustering is not used to directly assign epitope specificity, but rather to identify repertoire‐level patterns associated with a phenotype of interest. Clusters that are enriched in specific conditions can be interpreted as candidate antigen‐driven T cell responses, which can subsequently be linked to their cognate epitopes using bottom‐up annotation methods.

For example, in a yellow fever virus (YFV) vaccination study, a clustering‐based workflow was applied to longitudinal TCR repertoires collected before and after vaccination [53]. TCR sequences were grouped using ClusTCR, allowing the identification of clusters representing putative antigen‐driven responses. Comparing cluster abundances across timepoints, we observed a clear expansion of specific TCR clusters following vaccination, consistent with an antigen‐induced T cell response. Importantly, these expanded clusters were not defined by identical sequences, but rather by groups of related TCRs sharing common sequence features, highlighting the ability of clustering approaches to capture biologically meaningful variation beyond exact sequence matches. To further interpret these clusters, we integrated a bottom‐up annotation step using TCRex, which enabled the assignment of epitope specificity to the clustered TCRs. This analysis revealed that the expanded clusters were enriched for TCRs specific to the immunodominant YFV epitope LLWNGPMAV.

This study illustrates a key strength of clustering‐based top‐down approaches. By first identifying phenotype‐associated patterns at the repertoire level without prior knowledge, and subsequently linking them to antigen specificity through supervised models, it becomes possible to bridge discovery‐driven and mechanistic analyses. Within this framework, clustering serves as an intermediate layer that reduces sequence complexity, enhances statistical power and facilitates the identification of antigen‐driven T cell responses that would remain undetected when focusing on individual TCR sequences alone.

### 3.3 Convergence as a Less Biased Antigen‐Driven Repertoire Signature

While TCR enrichment and clustering provide valuable insight into antigen‐driven repertoire patterns, these approaches cannot fully distinguish true antigen‐driven selection from repertoire biases such as co‐occurring diseases, technical variation, cohort composition and background repertoire structure. For example, public clonotypes may arise not only from antigen exposure but also from biases in V(D)J recombination or thymic selection, leading to sequences that are frequently observed across individuals even in the absence of a shared immune response [54]. To further improve the identification of biologically meaningful signals, we can incorporate the concept of TCR convergence within our top‐down analyses.

Convergence refers to the independent generation of identical or highly similar TCR clonotypes through distinct recombination events [55, 56]. Due to the stochastic nature of V(D)J recombination, the probability of generating the same or highly similar TCR sequence multiple times independently is relatively low. Therefore, when such convergent sequences are repeatedly observed across individuals or within specific disease contexts, this suggests the presence of selective pressure, most likely driven by recognition of a shared antigen [57]. In this sense, convergence measures antigen‐driven selection relative to a baseline instead of simple sequence sharing or clonal expansion alone.

Convergence‐based approaches differ from general sequence matching or clustering methods because they distinguish antigen‐driven signals from background generation probabilities. By accounting for the local repertoire structure and sequence generation likelihood, these methods can discriminate between TCRs that are frequent due to recombination bias and those that are enriched through antigen‐specific selection. This makes convergence‐based analyses particularly valuable in settings where technical biases, uneven sampling or heterogeneous cohort composition would otherwise obscure true biological signals. Pioneering work by Pogorelyy et al. [58] introduced statistical frameworks that account for receptor generation probability when identifying antigen‐associated clonotypes, demonstrating that TCR sharing and convergence signals are strongly influenced by biases in V(D)J recombination. They subsequently extended this principle to single‐repertoire analysis to identify clusters of locally similar, expanded TCR sequences that occur more frequently than expected under a recombination‐based null model, enabling the detection of ongoing immune responses from a single repertoire snapshot without longitudinal sampling [59].

Building on this concept, our lab developed TRIASSIC [50], a top‐down method that quantifies TCR convergence, built on the fast and robust distance calculations implemented in ClusTCR. This allows scaling beyond a single repertoire to multi‐disease cohorts. The concept of convergence here is conceptually the same, a group of highly similar TCRs that cannot be explained by random chance. However, it now needs to account for a complex background recombination model which may feature repertoires from different individuals or even different studies. This setup allows discovery of convergent TCR clusters that are disease‐associated by comparing to a healthy or other representative cohort. The value of using convergence was demonstrated in our recent autoimmune arthritis study, in which we re‐analyzed 242 synovial fluid and tissue repertoires from 130 patients across 15 independent studies encompassing multiple forms of arthritis. These datasets exhibited substantial technical and cohort heterogeneity, posing a major challenge for conventional top‐down analyses. By applying TRIASSIC, we identified convergent TCRs that were significantly associated with disease phenotypes, despite the presence of strong confounding factors. This analysis identified clusters of convergent TCRs linked to specific disease features, including a known TRBV9‐associated cluster enriched in patients with HLA‐B27‐associated arthritis. Notably, these signals were not driven by simple sequence sharing or expansion alone, but by repeated emergence of similar TCRs, consistent with antigen‐driven selection. To further interpret these findings, we integrated bottom‐up annotation using DETECT, which suggested Epstein–Barr virus (EBV)‐specific reactivity within particular patient subgroups. Validation in independent datasets confirmed the reproducibility of these convergent, antigen‐associated TCR clusters.

Convergence‐based approaches thus provide a valuable complementary layer within top‐down analysis. By focusing on independently generated yet functionally similar TCRs, convergence offers a more specific indicator of antigen‐driven immune responses and enables the detection of biologically meaningful patterns that remain hidden to enrichment‐ or clustering‐based methods alone.

### 3.4 Top‐Down Challenges

Top‐down approaches provide a powerful framework for identifying antigen‐driven T cell responses directly from repertoire data without requiring prior knowledge of epitope specificity. However, this strength is accompanied by a number of important limitations. In practice, TCR repertoires are shaped not only by antigen exposure, but also by a complex interplay of technical factors, biological variation, and statistical constraints. This can generate signals that are indistinguishable from true antigen‐driven patterns, complicating interpretation and limiting reproducibility of the results.

#### 3.4.1 Technical Variability and Sequencing Bias

One of the most impactful challenges in top‐down analyses is the presence of technical variability introduced during sample processing and sequencing. Differences in library preparation methods, amplification strategies, sequencing platforms and even individual sequencing runs can introduce systematic biases that strongly affect repertoire composition [60]. In a large‐scale meta‐analysis of TCR repertoires across multiple chronic rheumatic diseases, we observed that variation in V‐ and J‐gene usage was dominated by technical factors rather than biological differences. Specific V‐gene segments such as TRBV12‐4 and TRBV12‐3 were strongly associated with sequencing methodology (10× Genomics vs. 5′ RACE), effectively separating samples based on protocol rather than disease state [48, 60]. Surprisingly, these effects exceeded differences observed between biologically distinct T cell subsets such as CD4^+^ and CD8^+^ repertoires [48]. Similar batch effects can arise even within studies using the same experimental protocol, where differences between sequencing runs introduce subtle but reproducible shifts in repertoire composition. These biases often persist despite standard quality control procedures, indicating that commonly applied normalization strategies are insufficient to fully correct for technical variation.

Because top‐down methods rely on detecting statistical differences between cohorts, they are particularly sensitive to such artifacts. Without careful correction, technical biases can be misinterpreted as disease‐associated signals, leading to false positive results. Addressing this challenge requires explicit modeling of background repertoire structure and the incorporation of bias‐aware normalization strategies, followed by validation across independent datasets.

#### 3.4.2 Biological Confounding and Cohort Heterogeneity

Beyond technical artifacts, biological variability also represents a major source of confounding in top‐down analyses. TCR repertoires are shaped by a wide range of factors unrelated to the phenotype of interest, including HLA genotype, infection history, age, and geographic background [61]. These variables can introduce strong signals that may overshadow disease‐specific effects.

Among these, HLA variation plays a central role. Because TCR recognition is inherently HLA‐restricted, differences in HLA allele frequencies between cohorts directly influence which epitopes can be presented and, consequently, which TCRs are selected and expanded [6, 7]. As a result, TCRs associated with specific HLA alleles may appear enriched in a given cohort, even in the absence of a disease‐specific immune response. This is particularly relevant in diseases with known HLA associations, such as HLA‐B27‐linked arthritis, where distinguishing between HLA‐driven and disease‐driven signals becomes challenging.

Similarly, variation in exposure to common pathogens can introduce strong confounding effects. Chronic infections such as cytomegalovirus (CMV) or Epstein–Barr virus (EBV) leave long‐lasting imprints on the TCR repertoire, including persistent clonal expansions and public TCR signatures [62, 63]. Differences in seroprevalence across populations, geographic regions or timepoints (e.g., pre‐ vs. post‐SARS‐CoV‐2 pandemic) can therefore lead to apparent cohort‐specific signals that are unrelated to the condition of interest. Because many top‐down approaches readily capture these dominant sources of variation, the identified TCR signatures may reflect underlying HLA distribution or infection history rather than disease‐specific immune responses.

Mitigating these effects requires careful cohort design, including matched controls, stratification by known confounders, and where possible, incorporation of HLA typing and serological data into the analysis. Taking into account these variables is essential for distinguishing true antigen‐driven signals from background biological variation.

#### 3.4.3 Statistical Challenges and False Discovery

Top‐down analyses inherently involve large‐scale statistical testing across highly complex and sparse datasets, which increases the risk of false positives and false negative hits. A typical sequenced repertoire may contain tens of thousands to millions of unique TCR sequences, many of which are observed only once. Identifying sequences or clusters that are significantly enriched in a cohort therefore requires extensive multiple testing correction. In addition, the probability of observing shared or similar TCR sequences across individuals is influenced by the underlying generation probability of those sequences. Certain TCRs are more likely to be generated due to biases in V(D)J recombination [64], leading to “public” clonotypes that are frequently observed even in the absence of shared antigen exposure. Without accounting for these generation probabilities, enrichment‐based methods may incorrectly link such sequences to antigen‐driven selection.

These challenges also include variability in sequencing depth and repertoire size across samples, which can significantly complicate frequency‐based comparisons. Together, these elements make it difficult to distinguish true biological signals from stochastic variation and background noise.

#### 3.4.4 Interpretation and Specificity Limitations

A fundamental limitation of top‐down approaches is that they do not directly measure antigen specificity. Instead, they infer antigen‐driven responses indirectly through patterns such as clonal expansion, sequence sharing, and convergence. While these signals are informative, they are not uniquely indicative of antigen recognition. For example, enrichment of a TCR sequence in a disease cohort does not necessarily imply that the sequence is specific to a disease‐relevant epitope. Similarly, clustering of related TCRs suggests shared structural features, but does not guarantee shared specificity. Even convergence, while a stronger indicator of selection, does not provide direct evidence of the underlying antigen.

Therefore, top‐down findings must be interpreted with caution and validated using complementary approaches. Integration with bottom‐up prediction models and experimental assays is often required to assign antigen specificity and establish biological relevance.

#### 3.4.5 Toward Robust Top‐Down Inference

Addressing these challenges requires methodological advances that explicitly account for both technical and biological confounding, while improving the specificity of the identified signals. One promising direction is the incorporation of models that consider sequence generation probabilities when evaluating enrichment [50]. By integrating such strategies with careful cohort design, cross‐dataset validation and complementary bottom‐up annotation, top‐down analyses can achieve greater robustness and biological interpretability. Ultimately, while top‐down approaches offer a powerful means of discovering antigen‐driven patterns in large‐scale repertoire data, successfully applying them depends on strict control of confounding factors and integration with other sources of information.

## 4 Integration of Multiple Data Modalities

While top‐down TCR analyses can identify candidate antigen‐driven TCRs, interpreting their biological and functional relevance often requires integration with other data modalities (Table 1). TCR sequence information alone provides limited insight into the cellular state, tissue context and environmental drivers of the immune response. By combining TCR repertoire data with gene expression profiles (RNA‐seq), spatial localization, microbial abundance, cytometric measurements and clinical metadata, it becomes possible to place TCR patterns within a broader immunological framework and link sequence‐level observations to functional immune phenotypes.

### 4.1 Multi‐Level Pattern Sharing

An important strategy is provided by network‐based integration methods such as Similarity Network Fusion (SNF) [65]. These approaches construct separate similarity networks for each data modality. For example, a TCR‐based network reflecting repertoire similarity between individuals, a transcriptomic network capturing gene expression similarity and a microbiome network based on microbial composition. Through an iterative fusion process, these networks are combined into a single unified graph that captures shared structures across modalities. In this framework, individuals or samples that cluster together in the fused network share consistent patterns across multiple biological layers. From a TCR‐centric point of view, this enables the identification of patient subgroups defined not only by repertoire similarity, but also by shared transcriptional programs, microbial environments and clinical features [66]. Such integrative clustering can reveal previously unrecognized disease subtypes or immune response patterns that would not be detectable from any single modality alone.

Latent‐variable methods provide a second option for highly interpretable frameworks for multimodal integration. Techniques such as (sparse) Canonical Correlation Analysis (CCA) [67, 68] and Partial Least Squares (PLS) [69] identify shared axes of variation between datasets by projecting them into a common latent space. In this context, TCR‐derived features, such as convergence scores, cluster membership or epitope enrichment signals can be explicitly modeled against transcriptomic, cytometric or microbiome‐derived features. This allows the identification of correlated latent components that capture coordinated variation between immune receptor repertoires and other biological systems. Sparse extensions such as sPLS further enhance interpretability by selecting a limited set of features that contribute most strongly to these shared components, thereby highlighting specific TCR clusters and molecular pathways associated with disease phenotypes. These approaches are powerful for generating mechanistic hypotheses, as they directly link TCR‐driven signals to functional readouts that can be validated experimentally [70].

### 4.2 Linking T Cell Phenotypes and Environmental Factors to the Repertoire

Multimodal integration also encompasses the combination of TCR data with transcriptomic profiles. The TCR‐centric multimodal framework STEGO [51], for example, combines TCR repertoire data with single‐cell or bulk RNA‐seq to jointly analyze clonal expansion and cellular phenotypes. In practice, STEGO identifies clusters of TCRs that are enriched within specific transcriptional states and then characterizes their associated gene expression signatures. This enables the identification of antigen‐driven T cell populations alongside their functional properties, such as effector, memory or exhausted phenotypes. In disease contexts, this approach provides insights not only into which TCRs are enriched, but also into how the corresponding T cells behave at the molecular level, thereby linking repertoire dynamics to functional roles in pathology or protection.

Integrating different data modalities is also incredibly important in complex diseases involving multiple interacting biological compartments. Crohn's disease (CD), for example, affects the gut in which the TCR repertoire of mucosal immunity reflects a balanced composition of diverse T cell subsets, required to maintain homeostasis with the microbiome. To investigate this environment, we analyzed the repertoire structure and cell phenotype distribution in monozygotic twins with Crohn's disease (CD) [71]. This setting provides a unique opportunity to identify genetic and environmental effects on the immune response and its T cell phenotypes. Initial top‐down repertoire‐level analyses included calculating TCR neighbor enrichment and convergence (TRIASSIC) in concordant (both twins affected) and discordant (only one twin affected) twins. The analyses showed that concordant twins shared more TCR sequences than discordant twins or healthy controls, suggesting a shared immune component.

To further characterize these signals, the top‐down results were combined with clustering (ClusTCR) of the convergent TCRs to detect broader patterns of antigen‐driven selection. This resulted in the identification of convergent TCR motifs across the repertoire. T cell subtype information was added to explore T cell phenotypes within the selected CD‐associated clusters. This revealed convergent clusters spanning mucosal‐associated invariant T cells, as well as previously uncharacterized TCRs, potentially linked to disease development and progression. This study proves that integration of top‐down convergence‐based analysis with clustering and phenotype annotation enables the detection of subtle but biologically meaningful repertoire patterns that are not captured by traditional enrichment‐based approaches alone.

Additionally, there is a direct and dynamic interaction between the T cell compartment and the gut microbiome, which plays a critical role in shaping local immune responses and disease outcomes. To explore these interactions, we developed the Adaptive Immune Receptor Repertoire‐Wide Association Study (AIRRWAS) framework, a top‐down method that integrates TCR repertoire features with microbial abundance profiles within a unified statistical framework [52]. This approach enables the identification of associations between convergent TCR clusters and specific bacterial genera. AIRRWAS was applied to the CD dataset, alongside cohorts of individuals with colorectal cancer (CRC) and healthy controls. This design allowed the identification of baseline TCR‐microbiome interactions associated with the healthy gut and their comparison across disease contexts to discover disease‐associated TCR‐microbiome interactions. The resulting baseline interactions were proven consistent across independent datasets, supporting their generalizability and robustness of the method. Experimental validation using microbial stimulation assays further confirmed that T cells carrying these TCRs responded specifically to the associated bacterial taxa, providing direct functional evidence for the inferred relationships.

Together, this illustrates how integrating TCR repertoire analysis with microbial and cellular information enables the identification of biologically meaningful interactions between immune receptors and environmental antigens. By combining multiple top‐down strategies with complementary data modalities, these integrative approaches move beyond purely correlative analyses and provide a more comprehensive framework for understanding immune‐microbiome interactions in health and disease.

### 4.3 Tracking Immune Exposure and Epidemiology

Lastly, integrated bottom‐up and top‐down approaches can be extended to the population level to study immune exposure and epidemiology. By combining TCR repertoire data with clinical metadata, serological information and additional omics layers, it becomes possible to track infection dynamics and immune responses across large cohorts. TCR signatures can capture population‐level patterns of immune exposure, offering a complementary perspective to traditional serology. When coupled with bottom‐up epitope annotation models, these signatures can be further refined and linked to specific antigens, forming a powerful iterative framework that bridges discovery and mechanistic interpretation.

This integrative strategy is used in several recent studies exploring the TCR repertoire in different settings. A first example is exploring the specificity and evolution of T cell responses during COVID‐19 [72]. In this study, bottom‐up epitope prediction models were first trained on MHC‐I‐presented SARS‐CoV‐2 epitopes, and subsequently applied to longitudinal CD8^+^ TCR repertoires from patients with varying COVID‐19 severity. These models enable the identification of SARS‐CoV‐2‐specific TCRs as well as cross‐reactive TCRs recognizing common coronavirus epitopes. Longitudinal analysis using these models revealed that both critical and non‐critical patients initially rely on cross‐reactive T cells targeting conserved coronavirus epitopes. However, only non‐critical patients rapidly develop a diverse and expanded repertoire of TCRs recognizing SARS‐CoV‐2‐specific epitopes, whereas critically ill individuals show delayed expansion and reduced diversity of these responses. These findings highlight how linking repertoire features to functional outcomes like disease severity can uncover clinically relevant immune dynamics, such as the timing, diversity and specificity of T cell responses, that are not easily captured by TCR sequencing alone.

Besides epidemiological applications, multimodal profiling of the TCR repertoire also enables extraction of overlapping biological signals within complex immune systems. By integrating multiple data layers, it becomes possible to delineate the processes that shape immune variation across individuals. We explored the relationship between different data modalities in a recent large‐scale multimodal study integrating immune cell phenotypes, TCR repertoires, transcriptomics, microbiome composition and clinical data from 394 individuals [66]. The results demonstrated that different modalities capture largely distinct aspects of human biology. Notably, different immune states and gut microbial “enterotypes” were found to be largely orthogonal, while the blood transcriptome acted as the only layer linking these systems. However, across modalities, inflammation emerged as a stable and consistent driver of variation. It is therefore important to capture the full immunotype rather than features in isolation to get a clear overview of all factors influencing the immunological state.

Similarly, in a study on chronic infections and immune aging, TCR repertoire sequencing was integrated with epigenetic profiling and CMV serostatus [62]. The results showed that frailty, characterized by functional decline, was associated with reduced diversity in the CD4^+^ TCR repertoire compared to healthy aging. Epigenetic clock signatures alone could not clearly distinguish between healthy aging and frailty. However, incorporating CMV serostatus revealed a significant increase in epigenetic aging in the CMV‐positive individuals. Integrating all data layers showed that frail individuals are characterized by decreased CD4^+^ diversity and increased fraction of CMV‐associated CD8^+^ T cells, while CMV‐positive individuals showed higher epigenetic aging compared to CMV‐negative individuals. These findings describe how chronic infections like CMV can reshape the immune state and confound epigenetic aging. It highlights the necessity of multimodal approaches to accurately represent immune dynamics and identify biologically meaningful biomarkers.

Overall, integrating TCR repertoire analysis with complementary data modalities provides a powerful and increasingly necessary framework for interpreting immune receptor diversity. By linking sequence‐level patterns to functional immune states, microbial exposures and clinical outcomes, these approaches enable a more comprehensive understanding of adaptive immunity. As multimodal datasets continue to grow in size and complexity, such integrative strategies will play a central role in translating TCR repertoire data into mechanistic insights and clinically relevant biomarkers.

## 5 Conclusions and Future Perspectives

The ability to map T cell receptors to their cognate epitopes remains one of the central challenges in modern immunology. Despite rapid advances in high‐throughput sequencing and computational modeling, the complexity of TCR‐pMHC recognition continues to limit our ability to accurately interpret adaptive immune responses. As highlighted throughout this review, this challenge arises not from a single bottleneck, but from the combined effects of extreme repertoire diversity, cross‐reactivity, HLA restriction, and the limited availability of high‐quality annotated data.

Within this landscape, bottom‐up and top‐down approaches have emerged as two complementary approaches for extracting actionable insights from TCR repertoire data. Bottom‐up models aim to resolve antigen specificity at the level of individual receptor‐epitope pairs, providing mechanistic insights into TCR recognition. These approaches have demonstrated strong performance for well‐characterized epitopes and have enabled the identification of epitope‐specific T cell responses in infection, cancer, and vaccination settings. However, their reliance on curated training datasets inherently limits their applicability, particularly for rare, poorly characterized, or entirely novel epitopes. In addition, challenges such as cross‐reactivity, biased training data, and the absence of reliable negative examples continue to constrain model generalizability.

In contrast, top‐down approaches shift the focus from individual receptors to the general structure and composition of TCR repertoires. By identifying patterns of clonal expansion, sequence similarity and convergence across individuals or cohorts, these methods enable the detection of antigen‐driven immune responses without requiring prior knowledge of epitope specificity. This discovery‐driven approach has proven particularly powerful for identifying disease‐associated TCR signatures and for capturing immune exposure history at the population level. At the same time, top‐down analyses face their own set of challenges, including sensitivity to technical variation, biological confounding and the indirect nature of identifying disease association but not epitope specificity.

A key insight emerging from recent work is that neither approach is sufficient on its own. Bottom‐up models provide specificity but are constrained by limited (low‐quality) data, while top‐down approaches provide much broader predictions but lack direct mechanistic resolution. Integrating these strategies offers a path forward by combining their complementary strengths. In such a framework, top‐down analyses can be used to identify candidate antigen‐driven TCRs or clusters within complex repertoires, after which bottom‐up models can assign putative epitope specificity [50, 53]. Thus, bottom‐up predictions can guide the interpretation of repertoire‐level patterns, enabling more targeted and biologically meaningful top‐down analyses. This iterative interplay between discovery and annotation represents a powerful paradigm for advancing TCR repertoire interpretation.

Efforts to bridge these approaches have also revealed several broader lessons about the TCR specificity prediction problem. First, data quality and composition often have a greater impact on model performance than the exact choice of algorithm. Reported advances in predictive accuracy could be traced to differences in dataset size, curation or evaluation strategy, rather than fundamental improvements in modeling [28, 45]. Second, HLA restriction is a central determinant of TCR specificity that is still insufficiently incorporated into most computational frameworks. Ignoring HLA context not only limits predictive performance but also contributes to confounding in both bottom‐up and top‐down analyses. Third, sequence similarity alone is an incomplete proxy for antigen specificity [17]. While motif‐based and clustering approaches capture important aspects of TCR recognition, they fail to account for the biological complexity of TCR‐pMHC interactions. Finally, the distinction between seen and unseen epitopes remains an important challenge, with most current models showing a substantial drop in performance when applied to novel epitopes [30].

The field will require continued methodological and experimental innovation. On the data side, expanding the scale, diversity, and quality of validated TCR‐epitope annotation datasets is essential. This includes increasing the availability of paired αβ chain data, improving standardization across experimental platforms, and incorporating more diverse antigen classes, such as tumor neoepitopes and microbiome‐derived peptides. The development of standardized benchmarking frameworks and community‐wide data sharing initiatives will further facilitate robust model evaluation, comparison, and improvement.

On the modeling side, future approaches are likely to move toward more integrative and biologically informed representations of TCR‐epitope interactions. Models that incorporate both TCR and epitope sequence information, as well as structural and physicochemical features, offer a promising direction for improving generalization to unseen epitopes. In parallel, advances in protein language models and large‐scale pretraining strategies may enable the extraction of transferable representations from unlabeled sequence data, partially alleviating the dependence on experimentally annotated interactions [37].

Equally important is the integration of TCR repertoire data with complementary modalities. As demonstrated by recent multimodal studies, combining TCR sequencing with transcriptomics, microbiome profiling, spatial data, and clinical metadata provides critical context for interpreting TCR repertoire composition. Such integrative approaches enable the linkage of sequence‐level patterns to cellular function, environmental exposure, and disease phenotype, moving beyond descriptive repertoire analysis toward a more systems‐level understanding of adaptive immunity.

From a translational perspective, these developments hold significant promise. Improved TCR‐epitope annotation has the potential to enable more precise immune monitoring in infections and diseases, to facilitate the identification of tumor‐reactive T cells in cancer immunotherapy [73, 74] and uncover autoreactive clones in autoimmune disorders [75]. In addition, repertoire‐derived biomarkers may provide sensitive and non‐invasive tools for disease diagnosis, prognosis, and treatment response prediction [18].

Looking forward, the field is likely to move toward unified frameworks that integrate bottom‐up specificity prediction, top‐down repertoire analysis and multimodal data integration within a single analytical pipeline. Such approaches will not only improve the accuracy and interpretability of TCR annotation, but also enable iterative refinement, where computational predictions guide experimental validation and vice versa. In this context, the boundary between discovery‐driven and hypothesis‐driven research becomes increasingly blurred. TCR‐epitope annotation represents a complex, high‐dimensional problem and progress will depend on the continued integration of complementary strategies, the development of more comprehensive and less biased datasets and the incorporation of biological context at multiple levels of resolution. By bridging molecular specificity with repertoire‐level patterns and population‐scale variation, the next generation of computational and experimental tools will provide a more complete and actionable understanding of T cell immunity.

## Funding

This work was supported by the iBOF Project “Modulating Immunity and the Microbiome for Effective CRC Immunotherapy” (MIMICRY) (K.L.), by fellowships from the Research Foundation Flanders (FWO) including 1SH6624N (V.V.D), 1SH3924N (F.A.) and 1S48819N (S.G.) as well as a grant for ELIXIR Belgium and iBOF‐MIMICRY. Additional support was provided by the Flemish Government “Onderzoeksprogramma Artificiële Intelligentie (AI) Vlaanderen” Program and University of Antwerp (BOF SEP 54073).

## Conflicts of Interest

P.M. and K.L. are shareholders and board members of ImmuneWatch BV.

## Data Availability

Data sharing not applicable to this article as no datasets were generated or analysed during the current study.

## References

[1] Z. Steier , E. J. Y. Kim , D. A. Aylard , and E. A. Robey , “The CD4 Versus CD8 T Cell Fate Decision: A Multiomics‐Informed Perspective,” Annual Review of Immunology 42 (2024): 235–258. 10.1146/annurev-immunol-083122-040929 38271641

[2] K. M. Ashby and K. A. Hogquist , “A Guide to Thymic Selection of T Cells,” Nature Reviews. Immunology 24 (2024): 103–117. 10.1038/s41577-023-00911-8 37464188

[3] K. Shah , A. Al‐Haidari , J. Sun , and J. U. Kazi , “T Cell Receptor (TCR) Signaling in Health and Disease,” Signal Transduction and Targeted Therapy 6 (2021): 412. 34897277 10.1038/s41392-021-00823-w PMC8666445

[4] M. Krogsgaard and M. M. Davis , “How T Cells ‘See’ Antigen,” Nature Immunology 6 (2005): 239–245. 15716973 10.1038/ni1173

[5] J. K. Kulski , S. Suzuki , and T. Shiina , “Human Leukocyte Antigen Super‐Locus: Nexus of Genomic Supergenes, SNPs, Indels, Transcripts, and Haplotypes,” Human Genome Variation 9 (2022): 49. 36543786 10.1038/s41439-022-00226-5 PMC9772353

[6] J. Rossjohn , S. Gras , J. J. Miles , S. J. Turner , D. I. Godfrey , and J. McCluskey , “T Cell Antigen Receptor Recognition of Antigen‐Presenting Molecules,” Annual Review of Immunology 33 (2015): 169–200. 10.1146/annurev-immunol-032414-112334 25493333

[7] E. Sharon , L. V. Sibener , A. Battle , H. B. Fraser , K. C. Garcia , and J. K. Pritchard , “Genetic Variation in MHC Proteins Is Associated With T Cell Receptor Expression Biases,” Nature Genetics 48 (2016): 995–1002. 27479906 10.1038/ng.3625 PMC5010864

[8] H. ElAbd , A. K. H. Mahdy , E. M. Wacker , et al., “T Cell Receptor Clonotypes Predict Human Leukocyte Antigen Allele Carriage and Antigen Exposure History,” Communications Biology 9 (2026): 50. 41530431 10.1038/s42003-025-09140-2 PMC12800166

[9] R. A. Mariuzza , P. Agnihotri , and J. Orban , “The Structural Basis of T‐Cell Receptor (TCR) Activation: An Enduring Enigma,” Journal of Biological Chemistry 295 (2020): 914–925. 31848223 10.1074/jbc.REV119.009411 PMC6983839

[10] P. Meysman , J. Barton , B. Bravi , et al., “Benchmarking Solutions to the T‐Cell Receptor Epitope Prediction Problem: IMMREP22 Workshop Report,” ImmunoInformatics 9 (2023): 100024.

[11] D. S. Fischer , Y. Wu , B. Schubert , and F. J. Theis , “Predicting Antigen Specificity of Single T Cells Based on TCR CDR3 Regions,” Molecular Systems Biology 16 (2020): e9416. 32779888 10.15252/msb.20199416 PMC7418512

[12] D. G. Schatz and Y. Ji , “Recombination Centres and the Orchestration of V(D)J Recombination,” Nature Reviews Immunology 11 (2011): 251–263. 10.1038/nri2941 21394103

[13] A. M. W. Thierry Mora , “Quantifying Lymphocyte Receptor Diversity,” *bioRxiv*, (2016), 10.1101/046870.

[14] M. K. Jenkins , H. H. Chu , J. B. McLachlan , and J. J. Moon , “On the Composition of the Preimmune Repertoire of T Cells Specific for Peptide‐Major Histocompatibility Complex Ligands,” Annual Review of Immunology 28 (2010): 275–294. 10.1146/annurev-immunol-030409-101253 20307209

[15] G. Petrova , A. Ferrante , and J. Gorski , “Cross‐Reactivity of T Cells and Its Role in the Immune System,” Critical Reviews in Immunology 32 (2012): 349–372. 23237510 10.1615/critrevimmunol.v32.i4.50 PMC3595599

[16] P. Dash , A. J. Fiore‐Gartland , T. Hertz , et al., “Quantifiable Predictive Features Define Epitope‐Specific T Cell Receptor Repertoires,” Nature 547 (2017): 89–93. 28636592 10.1038/nature22383 PMC5616171

[17] P. Meysman , N. de Neuter , S. Gielis , D. Bui Thi , B. Ogunjimi , and K. Laukens , “On the Viability of Unsupervised T‐Cell Receptor Sequence Clustering for Epitope Preference,” Bioinformatics 35 (2019): 1461–1468. 30247624 10.1093/bioinformatics/bty821

[18] R. A. Arnaout , E. T. L. Prak , N. Schwab , F. Rubelt , and Adaptive Immune Receptor Repertoire, C , “The Future of Blood Testing Is the Immunome,” Frontiers in Immunology 12 (2021): 626793. 33790897 10.3389/fimmu.2021.626793 PMC8005722

[19] R. O. Emerson , W. S. DeWitt , M. Vignali , et al., “Immunosequencing Identifies Signatures of Cytomegalovirus Exposure History and HLA‐Mediated Effects on the T Cell Repertoire,” Nature Genetics 49 (2017): 659–665. 28369038 10.1038/ng.3822

[20] K. Mayer‐Blackwell , S. Schattgen , L. Cohen‐Lavi , et al., “TCR Meta‐Clonotypes for Biomarker Discovery With tcrdist3 Enabled Identification of Public, HLA‐Restricted Clusters of SARS‐CoV‐2 TCRs,” eLife 10 (2021): e68605. 34845983 10.7554/eLife.68605 PMC8631793

[21] J. D. Altman , P. A. H. Moss , P. J. R. Goulder , et al., “Phenotypic Analysis of Antigen‐Specific T Lymphocytes,” Science 274 (1996): 94–96. 8810254 10.1126/science.274.5284.94

[22] A. Grifoni , D. Weiskopf , S. I. Ramirez , et al., “Targets of T Cell Responses to SARS‐CoV‐2 Coronavirus in Humans With COVID‐19 Disease and Unexposed Individuals,” Cell 181 (2020): 1489–1501.e15. 32473127 10.1016/j.cell.2020.05.015 PMC7237901

[23] J. A. Pai and A. T. Satpathy , “High‐Throughput and Single‐Cell T Cell Receptor Sequencing Technologies,” Nature Methods 18 (2021): 881–892. 34282327 10.1038/s41592-021-01201-8 PMC9345561

[24] A. V. Joglekar and G. Li , “T Cell Antigen Discovery,” Nature Methods 18 (2021): 873–880. 32632239 10.1038/s41592-020-0867-z

[25] D. Hudson , R. A. Fernandes , M. Basham , G. Ogg , and H. Koohy , “Can We Predict T Cell Specificity With Digital Biology and Machine Learning?,” Nature Reviews. Immunology 23 (2023): 511–521. 10.1038/s41577-023-00835-3 PMC9908307 36755161

[26] S. Valpione , P. A. Mundra , E. Galvani , et al., “The T Cell Receptor Repertoire of Tumor Infiltrating T Cells Is Predictive and Prognostic for Cancer Survival,” Nature Communications 12 (2021): 4098. 10.1038/s41467-021-24343-x PMC8253860 34215730

[27] P. Moris , J. de Pauw , A. Postovskaya , et al., “Current Challenges for Unseen‐Epitope TCR Interaction Prediction and a New Perspective Derived From Image Classification,” Briefings in Bioinformatics 22 (2021): bbaa318. 33346826 10.1093/bib/bbaa318 PMC8294552

[28] C. Dens , K. Laukens , W. Bittremieux , and P. Meysman , “The Pitfalls of Negative Data Bias for the T‐Cell Epitope Specificity Challenge,” Nature Machine Intelligence 5 (2023): 1060–1062.

[29] M. F. Jensen and M. Nielsen , “Enhancing TCR Specificity Predictions by Combined Pan‐ and Peptide‐Specific Training, Loss‐Scaling, and Sequence Similarity Integration,” eLife 12 (2024): RP93934. 38437160 10.7554/eLife.93934 PMC10942633

[30] M. Nielsen , A. Eugster , M. F. Jensen , et al., “Lessons Learned From the IMMREP23 TCR‐Epitope Prediction Challenge,” ImmunoInformatics 16 (2024): 100045.

[31] M. Shugay , D. V. Bagaev , I. V. Zvyagin , et al., “VDJdb: A Curated Database of T‐Cell Receptor Sequences With Known Antigen Specificity,” Nucleic Acids Research 46 (2018): D419–D427. 28977646 10.1093/nar/gkx760 PMC5753233

[32] N. Tickotsky , T. Sagiv , J. Prilusky , E. Shifrut , and N. Friedman , “McPAS‐TCR: A Manually Curated Catalogue of Pathology‐Associated T Cell Receptor Sequences,” Bioinformatics 33 (2017): 2924–2929. 28481982 10.1093/bioinformatics/btx286

[33] R. Vita , N. Blazeska , D. Marrama , et al., “The Immune Epitope Database (IEDB): 2024 Update,” Nucleic Acids Research 53 (2025): D436–D443. 39558162 10.1093/nar/gkae1092 PMC11701597

[34] S. Gielis , P. Moris , W. Bittremieux , et al., “Detection of Enriched T Cell Epitope Specificity in Full T Cell Receptor Sequence Repertoires,” Frontiers in Immunology 10 (2019): 2820. 31849987 10.3389/fimmu.2019.02820 PMC6896208

[35] J. Ostmeyer , S. Christley , I. T. Toby , and L. G. Cowell , “Biophysicochemical Motifs in T‐Cell Receptor Sequences Distinguish Repertoires From Tumor‐Infiltrating Lymphocyte and Adjacent Healthy Tissue,” Cancer Research 79 (2019): 1671–1680. 30622114 10.1158/0008-5472.CAN-18-2292 PMC6445742

[36] Y. Katayama and T. J. Kobayashi , “Comparative Study of Repertoire Classification Methods Reveals Data Efficiency of k‐Mer Feature Extraction,” Frontiers in Immunology 13 (2022): 797640. 35936014 10.3389/fimmu.2022.797640 PMC9346074

[37] K. Kinoshita and T. J. Kobayashi , “TCR Representation Learning With Protein Language Models: A Comprehensive Review,” International Immunology 38 (2026): 15–27. 40855636 10.1093/intimm/dxaf048 PMC12802949

[38] S. Valkiers , M. Van Houcke , K. Laukens , and P. Meysman , “ClusTCR: A Python Interface for Rapid Clustering of Large Sets of CDR3 Sequences With Unknown Antigen Specificity,” Bioinformatics 37 (2021): 4865–4867. 34132766 10.1093/bioinformatics/btab446

[39] “ImmuneWatch DETECT, Version 1.0. Developed by ImmuneWatch BV,” (2024), https://www.immunewatch.com/detect.

[40] S. Nolan , M. Vignali , M. Klinger , et al., “A Large‐Scale Database of T‐Cell Receptor Beta Sequences and Binding Associations From Natural and Synthetic Exposure to SARS‐CoV‐2,” Frontiers in Immunology 16 (2025): 1488851. 40034696 10.3389/fimmu.2025.1488851 PMC11873104

[41] J. Dean , R. O. Emerson , M. Vignali , et al., “Annotation of Pseudogenic Gene Segments by Massively Parallel Sequencing of Rearranged Lymphocyte Receptor Loci,” Genome Medicine 7 (2015): 123. 26596423 10.1186/s13073-015-0238-z PMC4657264

[42] S. Gielis , D. Flumens , S. van der Heijden , et al., “Analysis of Wilms' Tumor Protein 1 Specific TCR Repertoire in AML Patients Uncovers Higher Diversity in Patients in Remission Than in Relapsed,” Annals of Hematology 104 (2025): 317–333. 39259326 10.1007/s00277-024-05919-1 PMC11868354

[43] C. Dens , W. Bittremieux , F. Affaticati , K. Laukens , and P. Meysman , “Interpretable Deep Learning to Uncover the Molecular Binding Patterns Determining TCR–Epitope Interaction Predictions,” ImmunoInformatics 11 (2023): 100027.

[44] E. Jokinen , A. Dumitrescu , J. Huuhtanen , et al., “TCRconv: Predicting Recognition Between T Cell Receptors and Epitopes Using Contextualized Motifs,” Bioinformatics 39 (2023): btac788. 36477794 10.1093/bioinformatics/btac788 PMC9825763

[45] Y. Lu , Y. Wang , M. Xu , et al., “Assessment of Computational Methods in Predicting TCR–Epitope Binding Recognition,” Nature Methods 23 (2026): 248–259. 41315816 10.1038/s41592-025-02910-0 PMC12791011

[46] A. Mayer and C. G. Callan, Jr. , “Measures of Epitope Binding Degeneracy From T Cell Receptor Repertoires,” Proceedings of the National Academy of Sciences of the United States of America 120 (2023): e2213264120. 36649423 10.1073/pnas.2213264120 PMC9942805

[47] G. Croce , S. Bobisse , D. L. Moreno , et al., “Deep Learning Predictions of TCR‐Epitope Interactions Reveal Epitope‐Specific Chains in Dual Alpha T Cells,” Nature Communications 15 (2024): 3211. 10.1038/s41467-024-47461-8 PMC11016097 38615042

[48] S. Valkiers , A. Dams , M. Kuznetsova , et al., “Linking Myelin and Epstein‐Barr Virus Specific Immune Responses in Multiple Sclerosis: Insights From Integrated Public T Cell Receptor Repertoires,” *bioRxiv*, (2024), 10.1101/2024.10.23.619834.

[49] N. De Neuter , E. Bartholomeus , G. Elias , et al., “Memory CD4(+) T Cell Receptor Repertoire Data Mining as a Tool for Identifying Cytomegalovirus Serostatus,” Genes and Immunity 20 (2019): 255–260. 29904098 10.1038/s41435-018-0035-y

[50] M. K. Ha , V. Van Deuren , C. A. de Carvalho Fraga , et al., “Generation of a T Cell Receptor, Cytokine and Cell Repertoire Synovial Fluid Atlas to Define Commonalities and Dissimilarities Between Arthritic Diseases Through Systems Immunology Approaches,” *bioRxiv*, (2025), 10.1101/2025.01.10.632345.

[51] K. A. Mullan , M. Ha , S. Valkiers , et al., “T Cell Receptor‐Centric Perspective to Multimodal Single‐Cell Data Analysis,” Science Advances 10 (2024): eadr3196. 39612336 10.1126/sciadv.adr3196 PMC11606500

[52] R. Vandoren , M. Ha , V. Van Deuren , et al., “T Cell‐Microbiome Associations Captured Through T Cell Receptor Convergence Analysis,” *bioRxiv*, (2025), 10.1101/2025.09.30.679432.

[53] S. Valkiers , S. Gielis , V. M. L. Van Deuren , K. Laukens , and P. Meysman , “Clustering and Annotation of T Cell Receptor Repertoires,” Methods in Molecular Biology 2673 (2023): 33–51. 37258905 10.1007/978-1-0716-3239-0_3

[54] M. F. Quigley , H. Y. Greenaway , V. Venturi , et al., “Convergent Recombination Shapes the Clonotypic Landscape of the Naive T‐Cell Repertoire,” Proceedings of the National Academy of Sciences of the United States of America 107 (2010): 19414–19419. 20974936 10.1073/pnas.1010586107 PMC2984183

[55] M. Pan and B. Li , “T Cell Receptor Convergence is an Indicator of Antigen‐Specific T Cell Response in Cancer Immunotherapies,” eLife 11 (2022): e81952. 36350695 10.7554/eLife.81952 PMC9683788

[56] V. Venturi , D. A. Price , D. C. Douek , and M. P. Davenport , “The Molecular Basis for Public T‐Cell Responses?,” Nature Reviews Immunology 8 (2008): 231–238. 10.1038/nri2260 18301425

[57] T. J. Looney , D. Topacio‐Hall , G. Lowman , et al., “TCR Convergence in Individuals Treated With Immune Checkpoint Inhibition for Cancer,” Frontiers in Immunology 10 (2019): 2985. 31993050 10.3389/fimmu.2019.02985 PMC6962348

[58] M. V. Pogorelyy , A. A. Minervina , D. M. Chudakov , et al., “Method for Identification of Condition‐Associated Public Antigen Receptor Sequences,” eLife 7 (2018): e33050. 29533178 10.7554/eLife.33050 PMC5873893

[59] M. V. Pogorelyy , A. A. Minervina , M. Shugay , et al., “Detecting T Cell Receptors Involved in Immune Responses From Single Repertoire Snapshots,” PLoS Biology 17 (2019): e3000314. 31194732 10.1371/journal.pbio.3000314 PMC6592544

[60] P. Barennes , V. Quiniou , M. Shugay , et al., “Benchmarking of T Cell Receptor Repertoire Profiling Methods Reveals Large Systematic Biases,” Nature Biotechnology 39 (2021): 236–245. 10.1038/s41587-020-0656-3 32895550

[61] W. S. DeWitt, III , A. Smith , G. Schoch , J. A. Hansen , F. A. Matsen, IV , and P. Bradley , “Human T Cell Receptor Occurrence Patterns Encode Immune History, Genetic Background, and Receptor Specificity,” eLife 7 (2018): e38358. 30152754 10.7554/eLife.38358 PMC6162092

[62] M. Breëns , K. De Man , Y. Heylen , et al., “The T Cell Receptor Repertoire Captures Healthy Aging and CMV Independently From Epigenetic Clocks,” *bioRxiv*, (2026), 10.64898/2026.02.20.706960.

[63] L. R. Wedderburn , A. Patel , H. Varsani , and P. Woo , “The Developing Human Immune System: T‐Cell Receptor Repertoire of Children and Young Adults Shows a Wide Discrepancy in the Frequency of Persistent Oligoclonal T‐Cell Expansions,” Immunology 102 (2001): 301–309. 11298828 10.1046/j.1365-2567.2001.01194.x PMC1783177

[64] A. Murugan , T. Mora , A. M. Walczak , and C. G. Callan , “Statistical Inference of the Generation Probability of T‐Cell Receptors From Sequence Repertoires,” Proceedings of the National Academy of Sciences of the United States of America 109 (2012): 16161–16166. 22988065 10.1073/pnas.1212755109 PMC3479580

[65] B. Wang , A. M. Mezlini , F. Demir , et al., “Similarity Network Fusion for Aggregating Data Types on a Genomic Scale,” Nature Methods 11 (2014): 333–337. 24464287 10.1038/nmeth.2810

[66] F. Affaticati , M. K. Ha , T. Gehrmann , et al., “Bridging Immunotypes and Enterotypes Using a Systems Immunology Approach,” *bioRxiv*, (2024), 10.1101/2024.11.29.625344.

[67] H. Hotelling , “Relations Between Two Sets of Variates,” in Breakthroughs in Statistics. Series in Statistics, ed. S. Kotz, and N. L. Johnson (Springer, 1992), 10.1007/978-1-4612-4380-9_14.

[68] T. Rodosthenous , V. Shahrezaei , and M. Evangelou , “Integrating Multi‐OMICS Data Through Sparse Canonical Correlation Analysis for the Prediction of Complex Traits: A Comparison Study,” Bioinformatics 36 (2020): 4616–4625. 32437529 10.1093/bioinformatics/btaa530 PMC7750936

[69] H. Wold , “Estimation of Principal Components and Related Models by Iterative Least Squares,” in Multivariate Analysis, ed. P. R. Krishnajah (Academic Press, 1966), 391–420.

[70] S. Huang , K. Chaudhary , and L. X. Garmire , “More Is Better: Recent Progress in Multi‐Omics Data Integration Methods,” Frontiers in Genetics 8 (2017): 84. 28670325 10.3389/fgene.2017.00084 PMC5472696

[71] E. C. Brand , R. Vandoren , L. Lutter , et al., “Clonal Overlap and Convergent Clustering of T‐Cell Receptor Signatures in Crohn's Disease in Monozygotic Twins,” *bioRxiv*, (2025), 10.1101/2025.10.31.685913. PMC13414540 42243622

[72] A. Postovskaya , A. Vujkovic , T. de Block , et al., “Leveraging T‐Cell Receptor – Epitope Recognition Models to Disentangle Unique and Cross‐Reactive T‐Cell Response to SARS‐CoV‐2 During COVID‐19 Progression/Resolution,” Frontiers in Immunology 14 (2023): 1130876. 37325653 10.3389/fimmu.2023.1130876 PMC10264683

[73] Y. Nose , I. Figueiredo , K. Tuballes , et al., “TCR Sequencing in Cancer Immunology and Immunotherapy: What, When, Where, Why, and How,” Journal for Immunotherapy of Cancer 14 (2026): e013499. 41775434 10.1136/jitc-2025-013499 PMC12958967

[74] Y. Li , M. Nahas , D. Stephens , et al., “Circulating T‐Cell Receptor Repertoire for Cancer Early Detection,” npj Precision Oncology 9 (2025): 245. 40684046 10.1038/s41698-025-01036-y PMC12276287

[75] A. M. Mitchell and A. W. Michels , “T Cell Receptor Sequencing in Autoimmunity,” Journal of Life Sciences (Westlake Village) 2 (2020): 38–58. 10.36069/jols/20201203 PMC7757640 33364626

